# Supplementary material for: The Evolution and Characterization of the RNA Interference Pathways in Lophotrochozoa
Source: Genome Biol Evol. 2024 May 7;16(5):evae098. doi: 10.1093/gbe/evae098 (PMC11114477; doi:10.1093/gbe/evae098)
Supplement: evae098_Supplementary_Data [file evae098_supplementary_data.zip › Supplementary_materials.zip!/selected_miRNAs/NoGe_selected_miRNAs/known_miRNA_families_pdf_files/NMRB01000426.1_Notospermus_geniculatus_strain_Ushimado_scaffold426,_whole_genome_shotgun_sequence_13050.pdf]

Provisional ID	:	NMRB01000426.1_Notospermus_geniculatus_strain_Ushimado_scaffold426,_whole_genome_shotgun_sequence_13050
Score total	:	154367.9
Score for star read(s)	:	3.9
Score for read counts	:	154360.4
Score for mfe	:	0.6
Score for randfold	:	0
Score for cons. seed	:	3
Total read count	:	302783
Mature read count	:	302744
Loop read count	:	3
Star read count	:	36

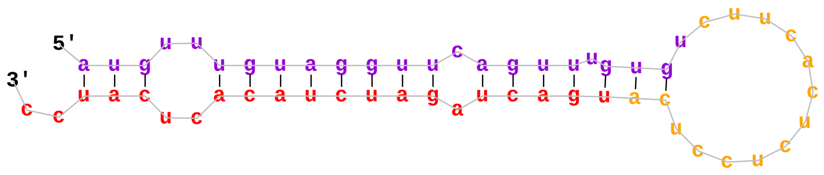

Genome coordinates: NMRB01000426.1 267076-267134

pre-miRNA sequence:
AUGUUUGUAGGUUCAGUUUGUGUAUGUUUGUAGGUUCAGUUUGUGUCUUCACUCUCCUCAUGACUAGAUCUACACUCAUCC

Mature sequence: UGACUAGAUCUACACUCAUCC

Aligned mature sequence of the *N. geniculatus* pre-miRNA with the top 5 hits from the BLAST search against the pre-miRNA mirGeneDB set:

```
                              ***************** *
Nge_Mir-279-o20               ugacuagaucuacacucaucc-
Hme-Mir-279-o32_3p            ugacuagaucuacacucauuga
Efe-Mir-279-o20_3p            ugacuagaucuacacucguccg
```
